# Caprylic Acid Inhibits High Mobility Group Box-1-Induced Mitochondrial Damage in Myocardial Tubes

**DOI:** 10.3390/ijms25158081

**Published:** 2024-07-24

**Authors:** Shota Nukaga, Rina Fujiwara-Tani, Ryoichi Nishida, Yoshihiro Miyagawa, Kei Goto, Isao Kawahara, Chie Nakashima, Kiyomu Fujii, Ruiko Ogata, Hitoshi Ohmori, Hiroki Kuniyasu

**Affiliations:** Department of Molecular Pathology, Nara Medical University, 840 Shijo-cho, Kashihara 634-8521, Nara, Japan; shota.nukaga@gmail.com (S.N.); g.m__r1@outlook.jp (R.N.); y.miya1103@gmail.com (Y.M.); ilgfgtk@gmail.com (K.G.); isao_kawahara@a011.broada.jp (I.K.); c-nakashima@naramed-u.ac.jp (C.N.); toto1999-dreamtheater2006-sms@nifty.com (K.F.); pkuma.og824@gmail.com (R.O.); brahmus73@hotmail.com (H.O.)

**Keywords:** medium-chain fatty acid, caprylic acid, cachexia, myocardial damage, mitochondria, HMGB1

## Abstract

Myocardial damage significantly impacts the prognosis of patients with cancer; however, the mechanisms of myocardial damage induced by cancer and its treatment remain unknown. We previously reported that medium-chain fatty acids (MCFAs) improve cancer-induced myocardial damage but did not evaluate the differences in effect according to MCFA type. Therefore, this study investigated the role of inflammatory cytokines in cancer-induced myocardial damage and the effects of three types of MCFAs (caprylic acid [C8], capric acid [C10], and lauric acid [C12]). In a mouse model, the C8 diet showed a greater effect on improving myocardial damage compared with C10 and C12 diets. Myocardial tubes differentiated from H9C2 cardiomyoblasts demonstrated increased mitochondrial oxidative stress, decreased membrane potential and mitochondrial volume, and inhibited myocardial tube differentiation following treatment with high-mobility group box-1 (HMGB1) but not interleukin-6 and tumor necrosis factor-α cytokines. However, HMGB1 treatment combined with C8 improved HMGB1-induced mitochondrial damage, enhanced autophagy, and increased mitochondrial biogenesis and maturation. However, these effects were only partial when combined with beta-hydroxybutyrate, a C8 metabolite. Thus, HMGB1 may play an important role in cancer-related myocardial damage. C8 counteracts HMGB1’s effects and improves cancer-related myocardial damage. Further clinical studies are required to investigate the effects of C8.

## 1. Introduction

Cancer cachexia occurs in approximately 80% of patients with advanced cancer [1], and heart failure is the second-leading cause of death in patients after death from cancer itself [2,3]. Myocardial damage makes it difficult to continue treatment and worsens life prognosis in patients with cancer [4]. Moreover, cancer survivors have a >1.5-fold increased risk of heart failure compared with patients without cancer [5]. Therefore, elucidating the mechanism of cancer-related myocardial damage and developing methods to ameliorate it are important challenges in cancer treatment.

Myocardial disorders in patients with cancer secondary to anticancer drug treatments are called cancer therapeutic-related cardiac dysfunction (CTRCD) [6,7,8]. We previously showed that the induction of ferroptosis associated with iron deposition in the myocardium plays an important role in CTRCD [9,10]. Moreover, in mouse models of cachexia, cancer can cause myocardial dysfunction even without anticancer-drug use [11]. Similarly, abnormal cardiac function has been observed even in patients with cancer cachexia who have not received anticancer drug treatment [12]. One cause of cancer-related myocardial damage may be energy metabolism disorders due to mitochondrial dysfunction, as mitochondrial uncoupling and reduced ATP production have been reported [6,13,14].

In cancer cachexia, inflammatory cytokines such as interleukin (IL)-1, tumor necrosis factor (TNF)-α, IL-6, IL-8, and high-motility group box-1 (HMGB1) promote catabolism through systemic inflammation, resulting in tissue damage [15,16]. HMGB1 is overexpressed in many cancers, including gastric and colon cancer, and promotes cell proliferation, invasion, and metastasis [17]. It also suppresses monocyte and antitumor immunity [17]. In colon carcinogenesis, HMGB1 expression is increased in the mucosa, and HMGB1 blood levels are correlated with cancer progression [18]. The blood concentration of HMGB1, elevated in patients with cachexia, is associated with TNFα concentration and is well correlated with sarcopenia in skeletal muscle cancer [16]. HMGB1 binds to receptors for advanced glycation end products (RAGEs) and toll-like receptor-4 (TLR4) in skeletal muscle, promoting autophagy and leading to atrophy [19]. HMGB1 inhibition reduces myocardial injury [18,19,20,21,22].

The importance of dietary interventions in cancer-related myocardial damage has attracted research attention [21,22]. Effective food nutrients include polyphenols, allicin, lycopene, polyunsaturated fatty acids, amino acids, coenzyme Q10, and trace elements [9,22]. We have previously reported the effect of lauric acid (C12), a medium-chain fatty acid (MCFA), on cancer-related myocardial damage in a mouse model [11]. C12 improves myocardial energy metabolism and has antitumor effects [11,23]. However, we observed no significant improvement in myocardial maturity.

In the present study, we compared the effects of C12 and other MCFAs, including caprylic acid (C8) and capric acid (C10), in a mouse cachexia model, as well as their effects on inflammatory cytokine-induced myocardial damage.

## 2. Results

### 2.1. Effects of MCFAs in a CT26 Mouse Cachexia Model

**Figure 1:**
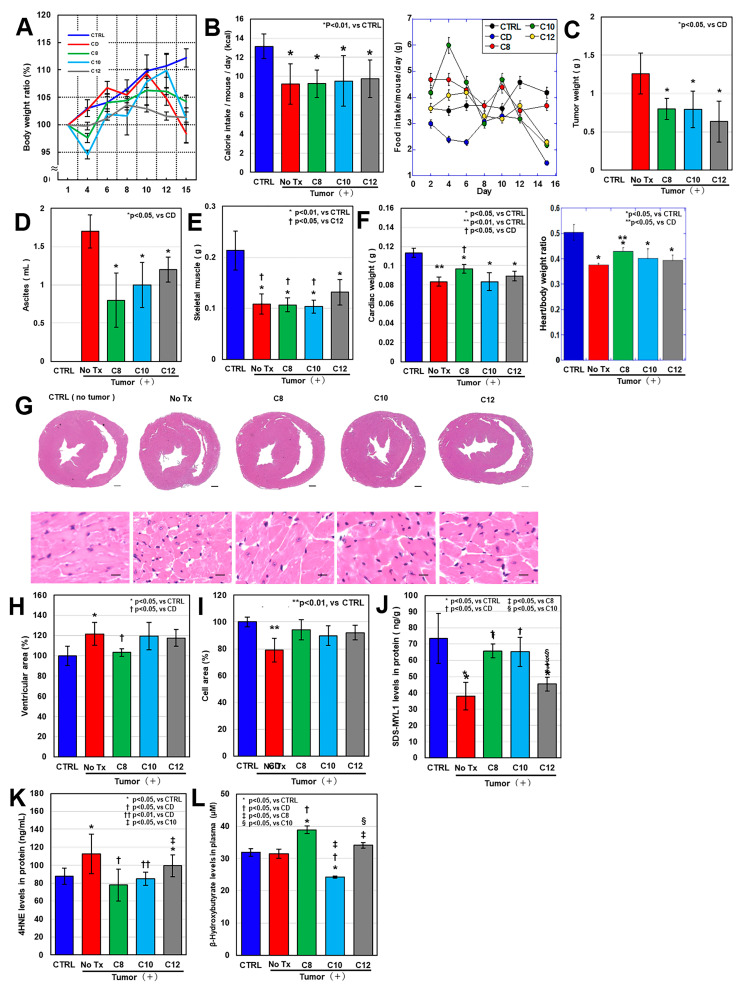
MCFA effects on myocardial damage in a mouse cachexia model. Male BALB/c mice (5 weeks old, three mice per group) were intraperitoneally inoculated with syngeneic CT26 mouse colon cancer cells (1 × 10^6^) to create a cachexia model [26]. C8, C10, and C12 were mixed with standard diet CE-2 at 2% *w*/*w* and provided ad libitum. The mice were euthanized after 2 weeks. (**A**) Body weight changes. (**B**) Calorie intake. (Right panel) Time course of food intake. (**C**) Tumor weight. (**D**) Ascites. (**E**) QCM weight. (**F**) Heart weights. (Right panel) Heart/body weight ratio. (**G**) Heart-cut surfaces and left ventricular tissue. Scale bars: cut surface, 300 μm; tissue, 10 μm. (**H**) Left ventricular lumen. (**I**) Left ventricular cardiomyocyte area. (**J**) Myocardial SDS-MYL1 staining. (**K**) Myocardial 4HNE. (**L**) Plasma ketone bodies (βHBA). Error bars: standard deviation from three mice. Statistical differences were calculated using analysis of variance with Bonferroni correction. C8, caprylic acid; C10, capric acid; C12, lauric acid; QCM, quadriceps femoris muscle; MCFA, medium chain fatty acid; No Tx, no treatment; SDS-MYL1, sodium dodecyl sulfate-soluble myosin light chain-1; 4HNE, 4-hydroxy-2-nonenal; βHBA, β-hydroxybutyric acid.

We used our previously established mouse model of cachexia [11,23] to examine the effects of three types of MCFAs on tumor and cardiac muscle. Five-week-old male BALB/c mice were inoculated intraperitoneally with syngeneic CT26 colon cancer cells and fed three types of MCFA at 2% each in a standard diet. Figure 1A shows the changes in mouse body weight. At the time of euthanasia (day 15), the body weights of all four tumor-bearing groups were lower than those of the non-tumor group (control), and no change was observed in the three types of MCFAs. Calorie intake was lower in all four tumor-bearing groups compared with the control group. (Figure 1B). Tumor weight and ascites were reduced in all MCFA-treated groups, although we observed no significant differences between the groups (Figure 1C,D). The weight of the quadriceps femoris muscle (QCM) was lower in all four tumor-bearing groups than in the control group. The QCM weights in the C8 and C10 groups did not differ from those in the CD (control diet) group; however, the C12 group showed a partial recovery of approximately 15% (Figure 1E). In contrast, heart weight was lower in all four tumor-bearing groups than in the control group, but only C8 showed a recovery of approximately 10% (Figure 1F). A comparison in terms of the heart weight/body weight ratio showed recovery only in C8 (Figure 1F right).

An examination of the morphological and biochemical changes in the myocardium (Figure 1G–K) revealed an increased left ventricular luminal area in the three tumor-loaded groups, except for the C8 group, which indicated left ventricular dilation. In contrast, the C8 group was equivalent to that of the control group (Figure 1H). The myocardial cell area decreased in the control group, indicating cardiac cell atrophy. In contrast, the levels in the MCFA group were equivalent to those in the control group (Figure 1I). The levels of sodium dodecyl sulfate-soluble myosin light chain-1 (SDS-MYL1), which indicate myosin-integrated MYL1 and correlates with muscle maturation [11,23,24,25], were decreased in the CD and C12 groups but were equivalent to the levels of the control group in the C8 and C10 groups (Figure 1J). In addition, myocardial lipid peroxidation (4-hydroxynonenal, 4HNE) increased in the CD group, normalized in the C8 and C10 groups, and it only partially recovered in the C12 group (Figure 1K).

As MCFAs reportedly generate ketone bodies through metabolism [26], we measured plasma β-hydroxybutyric acid (βHBA) concentrations (Figure 1L). The results revealed the highest βHBA increase in the C8 group, followed by the C12 group. Conversely, the βHBA level was decreased in C10. Thus, in a mouse cachexia model, C12 showed a restorative effect on skeletal muscle, while C8 helped restore cardiac muscle atrophy. In particular, we observed ketone body production with C8.

### 2.2. Effects of Inflammatory Cytokines on Myocardial Tube Differentiation in Cardiomyoblasts

**Figure 2:**
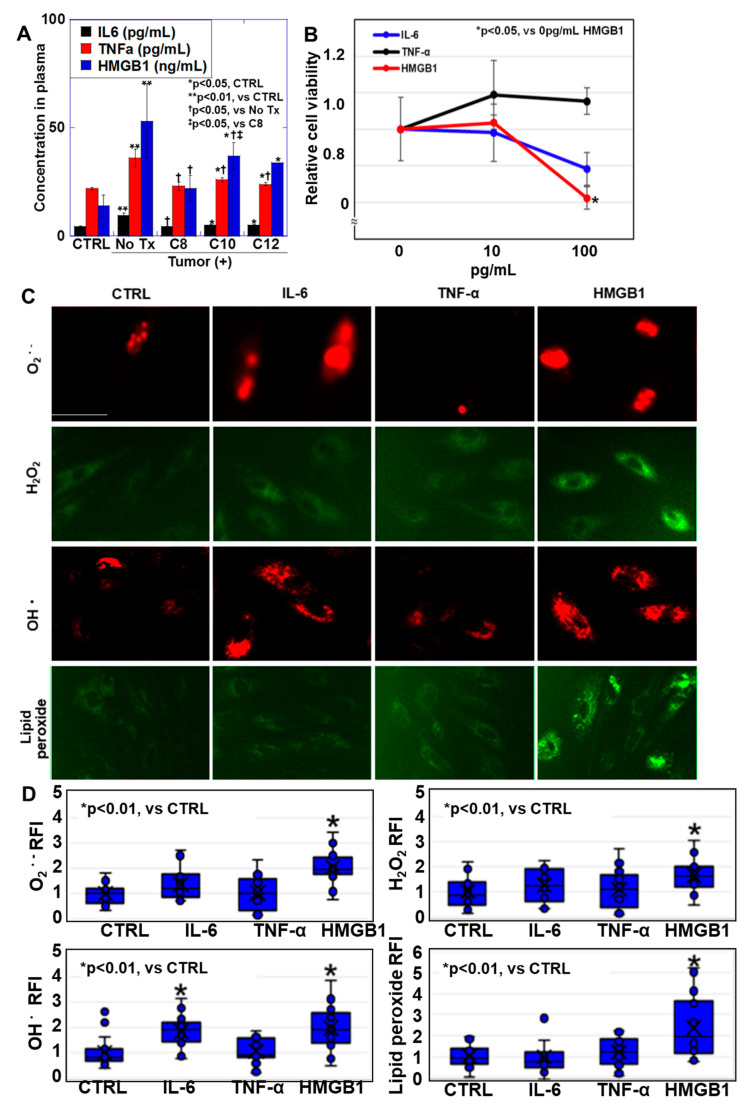

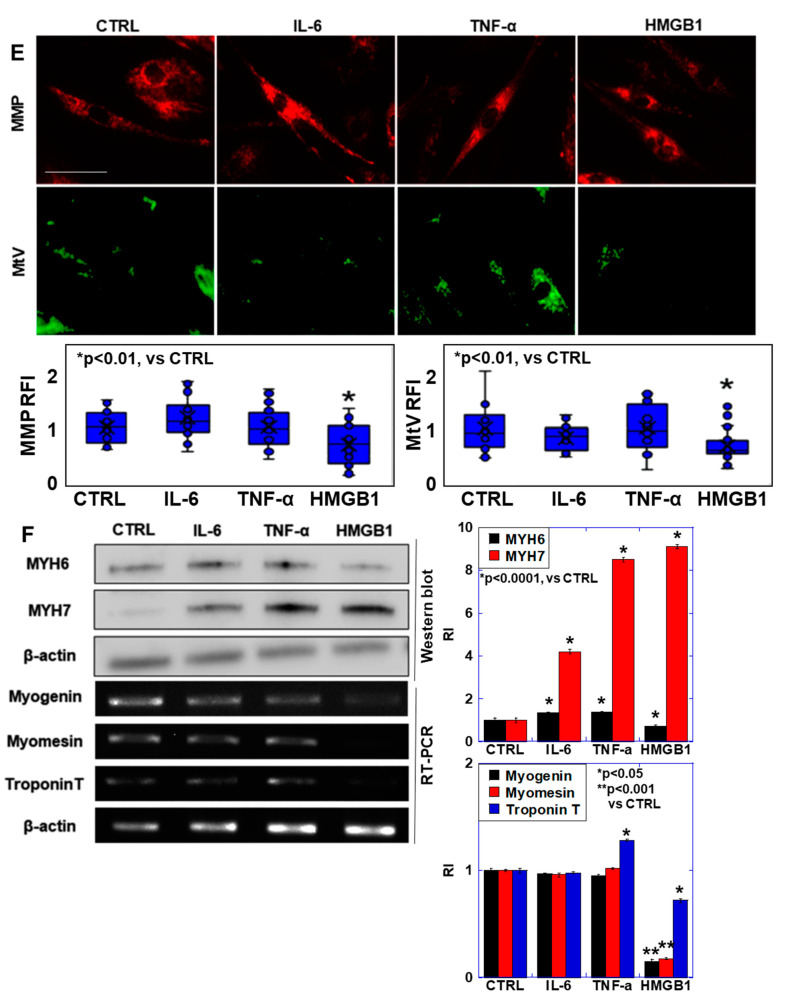
Effects of inflammatory cytokines on myocardial tube formation. (**A**) Plasma inflammatory cytokine concentrations in a mouse cachexia model. H9C2 rat cardiomyoblasts were cultured in Dulbecco’s modified Eagle medium (DMEM) containing 10% fetal bovine serum (FBS) and 1 μM retinoic acid for 48 h to induce differentiation into myocardial tubes and then treated with inflammatory cytokines (IL-6 (10 pg/mL), TNF-α (100 pg/mL), and HMGB1 (100 ng/mL)) for 48 h. (**B**) Effects of inflammatory cytokines on myocardial tube growth. (**C**) Mitochondrial oxidative stress in myocardial tubes. (**D**) Semi-quantification of panel C. (**E**) MMP and MtV. (Lower panel) Semi-quantification of panel C. (**F**) Myocardial tube differentiation. (Right panels) Semi-quantification of the left panel. Error bars, standard deviation of three independent trials, or 10 cases. Statistical differences were calculated using analysis of variance with the Bonferroni correction. C8, caprylic acid; C10, capric acid; C12, lauric acid; IL6, interleukin-6; TNFα, tumor necrosis factor-α; HMGB1, high-mobility group box-1; MMP, mitochondrial membrane potential; MtV, mitochondrial volume; MYH, myosin heavy chain; RFI, relative fluorescent intensity.

Inflammatory cytokines play important roles in cachexia [15]. The measurement of their blood concentrations in our mouse cachexia model (Figure 2A) revealed increased IL6, TNFα, and HMGB1 concentrations in the CD group. In contrast, the levels were higher in the MCFA group than those in the control group but lower than those in the CD group. The levels in the C8 group were lower than those in the C10 and C12 groups. To examine the effect of inflammatory cytokines on myocardial tubes, H9C2 cardiomyoblasts were induced to differentiate into myocardial tubes and then treated with inflammatory cytokines.

Our examination of the effects of inflammatory cytokines on myocardial tube proliferation (Figure 2B) revealed concentration-dependent inhibition of proliferation by HMGB1 but not IL6 and TNFα. Our evaluation of the effects of inflammatory cytokines on mitochondrial oxidative stress (Figure 2C,D) demonstrated that HMGB1 increased superoxide, hydrogen peroxide, hydroxyl radical, and lipid peroxide levels. In contrast, IL6 increased only hydroxyl radical levels. TNFα did not increase any reactive oxygen species (ROS) levels. We observed that IL6 and TNFα showed no significant changes in mitochondrial membrane potential (MMP) and mitochondrial volume compared with the control (Figure 2E). However, only HMGB1 decreased MMP and mitochondrial volume. An investigation of the effect of inflammatory cytokines on the expression of differentiation markers in the myocardial tubes (Figure 2F) showed that IL6 and TNFα increased MYH6 (adult expression, mature type) expression, while HMGB1 decreased its expression. In contrast, all cytokines increased MYH7 (fetal expression, immature type) expression, with HMGB1 inducing the largest differences. Furthermore, myogenin, myomesin, and troponin T all decreased with HMGB1, whereas troponin T increased with TNFα. Thus, among the three inflammatory cytokines, HMGB1 damages myocardial mitochondria and inhibits myocardial tube differentiation.

### 2.3. Effects of C8 and βHBA on HMGB1-Induced Myocardial Tube Damage

**Figure 3:**
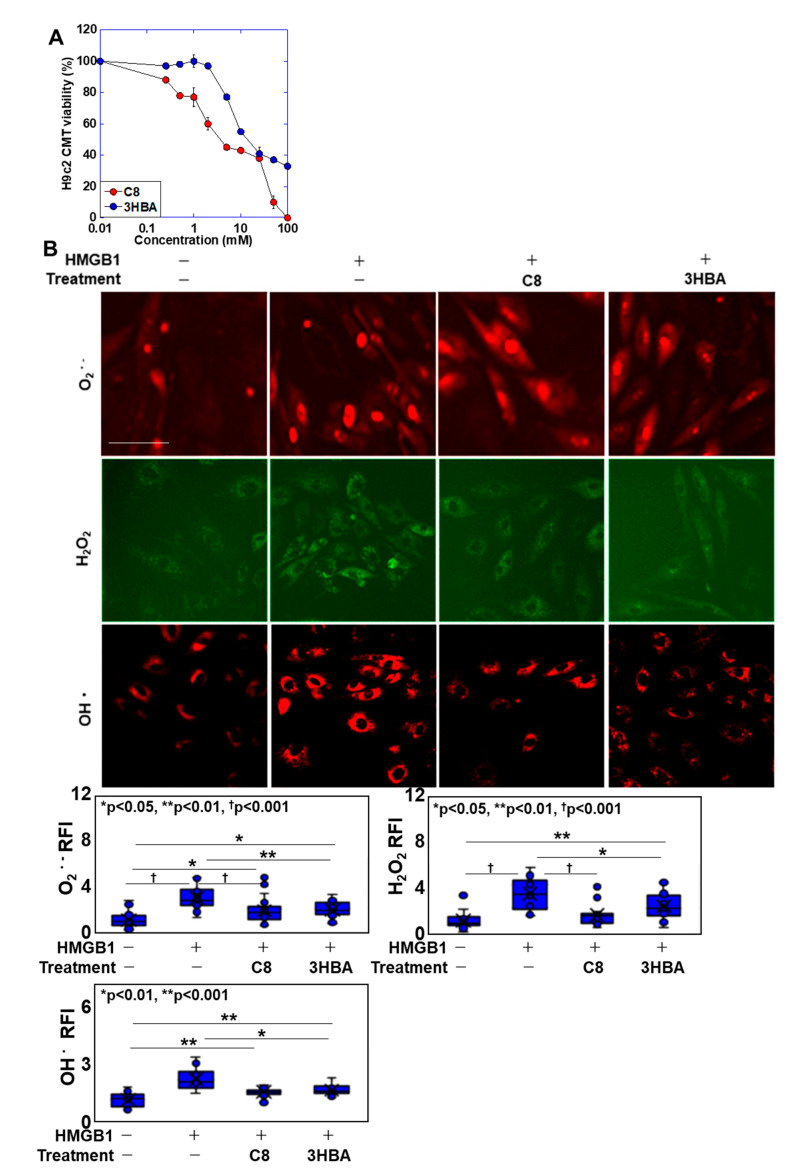

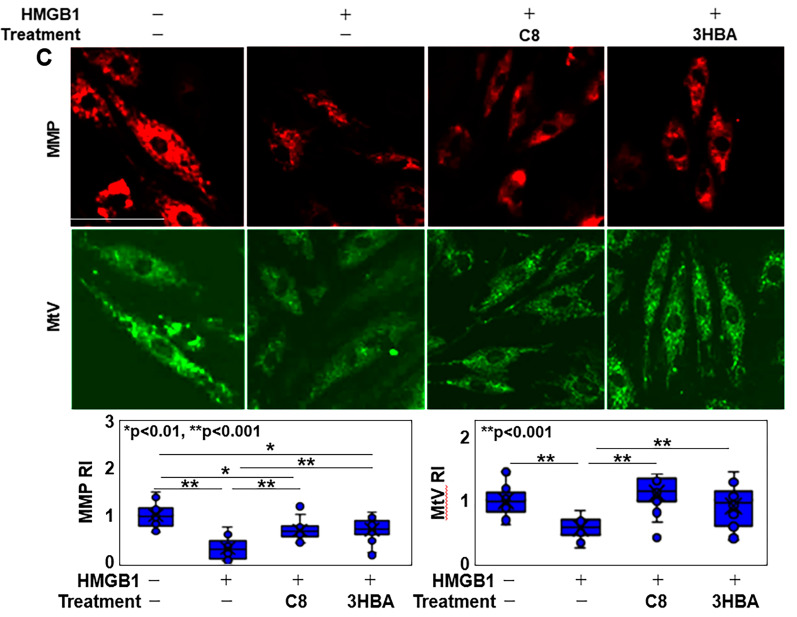
Effects of C8 and βHBA on HMGB1-induced myocardial tube injury. (**A**) Effects of C8 or βHBA on myocardial tube proliferation. (**B**,**C**) H9C2 rat cardiomyoblasts were induced to differentiate into myocardial tubes and then treated with HMGB1 (100 ng/mL) and/or C8 (0.25 mM) or βHBA (2 mM). (**B**) Mitochondrial oxidative stress in myocardial tubes. (**C**) Myocardial tube MMP and MtV. Error bars, standard deviation of three independent trials, or 10 cases. Statistical differences were calculated using analysis of variance with the Bonferroni correction. C8, caprylic acid; βHBA, β-hydroxybutyric acidHMGB1, high mobility group box-1; MMP, mitochondrial membrane potential; MtV, mitochondrial volume; RI, relative intensity.

Because C8 showed protective effects on the myocardium during cachexia in the above experiments, we investigated its mechanism of action in an in vitro system (Figure 3). In the mouse model, C8 produced βHBA; therefore, we also investigated the effects of βHBA. In addition, we treated H9C2 cells with HMGB1, which showed the strongest cardiomyocyte-damaging effects among the inflammatory cytokines. An investigation of the effects of C8 and βHBA on cell proliferation (Figure 3A) showed that both compounds inhibited cell proliferation in a concentration-dependent manner. We then treated the cells with C8 at 0.25 mM and βHBA at 2 mM, which are equivalent to the inhibitory concentrations causing 20% mortality (IC20) values, and investigated their effects on mitochondrial ROS enhancement by HMGB1 (Figure 3B). HMGB1 increased the levels of superoxide, hydrogen peroxide, and hydroxyl radicals by approximately threefold. In contrast, all ROS levels were restored to one-third when combined with C8 and to one-half when combined with βHBA. MMP was reduced to 30% by HMGB1 but was restored to 80% of the control value by C8 or βHBA (Figure 3C). Furthermore, mitochondrial volume was reduced to 60% by HMGB1 but was restored to the normal volume by C8 or βHBA. Thus, C8 and βHBA suppressed HMGB1-induced mitochondrial ROS production and the reduction in mitochondrial volume and MMP.

### 2.4. Effects of C8 and βHBA on HMGB1-Induced Myocardial Tube Quality Control

**Figure 4:**
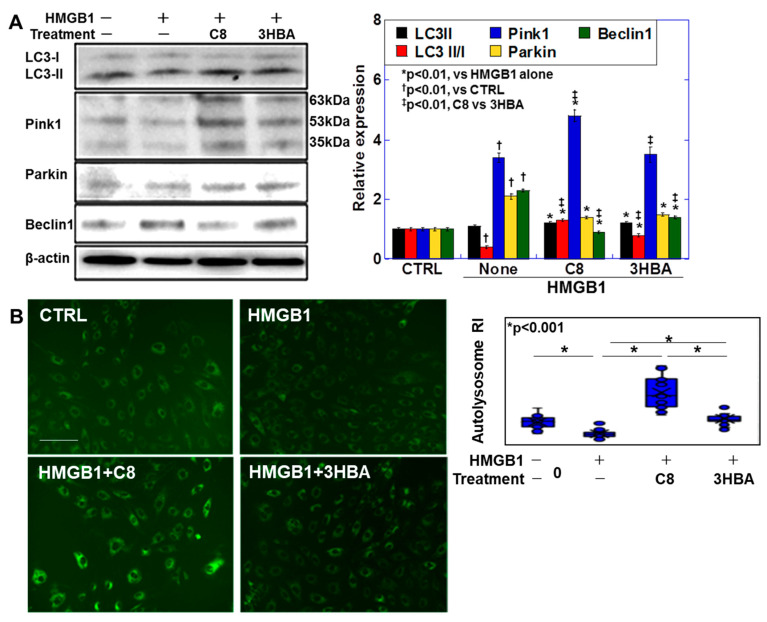

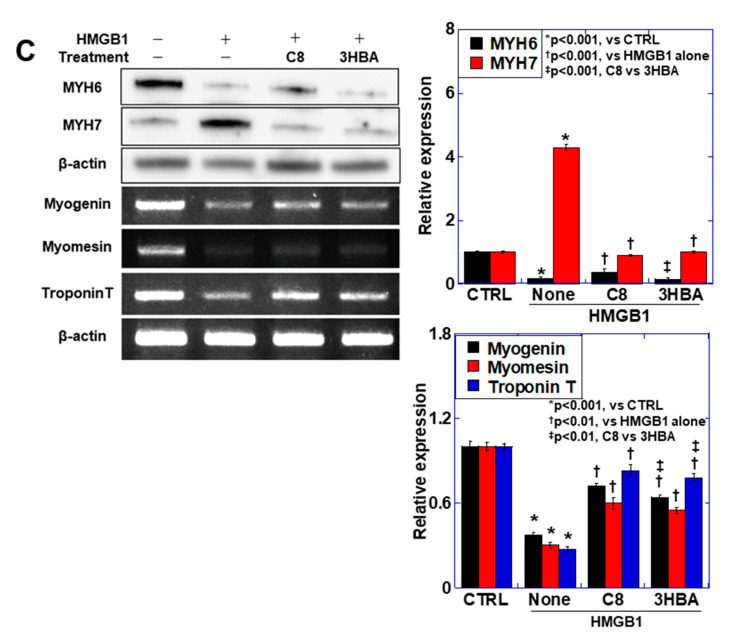
Effects of C8 and βHBA on the impairment of myocardial tube differentiation by HMGB1. (**A**) Autophagy-related protein levels. (**B**) Autophagy detected by DAL Green. (**C**) Myocardial tube differentiation. Error bars, standard deviation of three independent trials, or 10 cases. Statistical differences were calculated using analysis of variance with Bonferroni correction. C8, caprylic acid; βHBA, β-hydroxybutyric acidHMGB1, high mobility group box-1; LC, microtubule-associated protein 1A/1B-light chain; PINK1, PTEN-induced kinase 1; MMP, mitochondrial membrane potential; MtV, mitochondrial volume; MYH, myosin heavy chain; RI, relative intensity.

Next, we examined the effects of HMGB1 on myocardial tube quality control, as well as the potential protective role of C8 and βHBA against these effects. First, we observed that HMGB1 increased the levels of the autophagy-related proteins light-chain 3 (LC3-I) and BECLIN1 but not LC3-II and decreased the LC3-II/I ratio (Figure 4A). However, C8 and βHBA increased the LC3-II level and the LC-3II/I ratio. In addition, HMGB1 increased the levels of the mitophagy-related proteins PTEN-induced kinase 1 (PINK1) and parkin. C8 treatment further increased PINK1 levels. DALGreen staining, which was used to examine the actual state of autophagy by detecting autolysosomes (Figure 4B), revealed that HMGB1 reduced autolysosome counts by 40%, whereas the combined use of C8 increased them by 2.5-fold. In contrast, the combined use of βHBA restored autolysosomes to normal levels. Furthermore, our investigation of myocardial tube maturity (Figure 4C) demonstrated that HMGB1 reduced MYH6 expression by 20% and increased MYH7 expression by 480%. In contrast, C8 partially increased MYH6 expression and normalized MYH7 expression. The combination of βHBA did not improve MYH6 but normalized MYH7 expression levels. HMGB1 reduced myogenin, myomesin, and troponin T levels by approximately 40%. In contrast, the combination of C8 or βHBA partially restored the expression of these differentiation markers.

Thus, HMGB1 inhibited myocardial-tube quality control and differentiation, resulting in increased myocardial immaturity. In contrast, C8 and βHBA inhibited these effects, with C8 showing stronger inhibition.

### 2.5. Effects of C8 and βHBA on Mitochondrial Maturity

**Figure 5:**
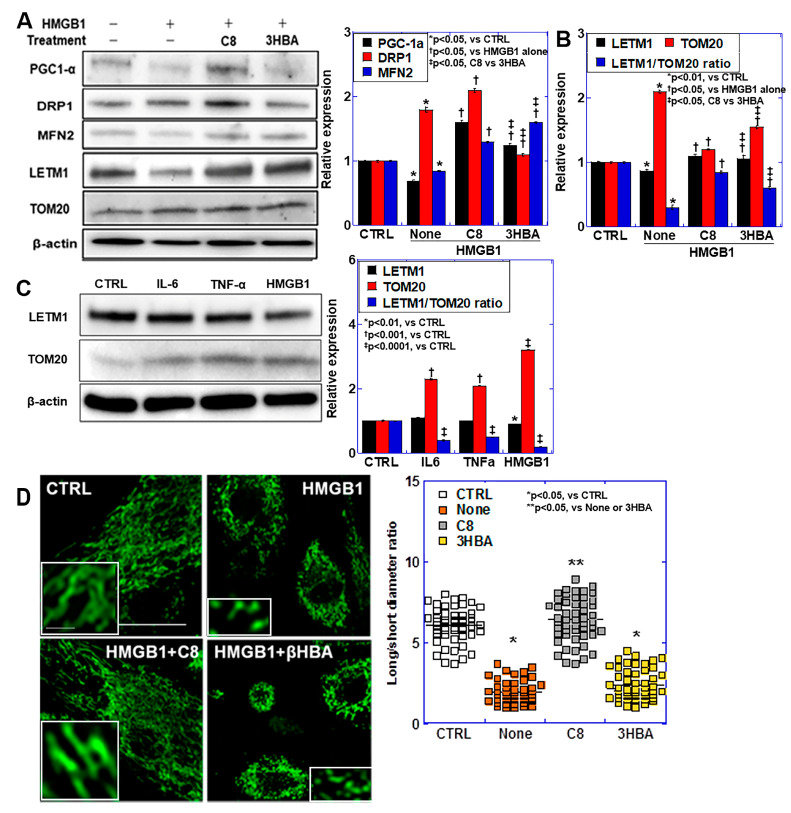
Effects of C8 and βHBA on HMGB1-induced mitochondrial maturation impairment. (**A**) Changes in mitochondrial biogenesis markers. (**B**) Changes in the inner (LETM1) and outer (TOM20) mitochondrial membrane. (**C**) Inflammatory cytokine-induced changes in LETM1 and TOM20. (**D**) Mitochondrial morphology. Scale bar, 20 μm. Inset, images with high-power solution. Scale bar, 1 μm. Right panel: mitochondrial long/short diameter ratios. Measured for 100 mitochondria. Scale bar, 5 μm. Error bars, standard deviation of three independent trials, or 10 cases. Statistical differences were calculated using analysis of variance with Bonferroni correction. C8, caprylic acid; βHBA, β-hydroxybutyric acid; HMGB1, high mobility group box-1; PGC1α, peroxisome proliferator-activated receptor γ coactivator-1α; DRP1, dynamin-related protein 1; MFN2, mitofusin-2; LETM1, leucine-containing zipper and EF-hand transmembrane protein 1; TOM20, translocase of the outer membrane-20; IL6, interleukin-6; TNFα, tumor necrosis factor-α.

As shown above, HMGB1 suppressed quality controls of myocardial tube and the mitochondria. Finally, we then examined the effect of C8 and βHBA against HMGB1-induced mitochondrial immaturity (Figure 5). As markers of mitochondrial maturity, we examined the protein levels of peroxisome proliferator-activated receptors gamma coactivator-1 alpha (PGC1α), which promotes mitochondrial biogenesis; dynamin-related protein-1 (DRP1), which promotes mitochondrial fusion; and mitofusin-2 (MFN2), which promotes mitochondrial fusion (Figure 5A). HMGB1 decreased PGC1α and MFN2 and increased DRP. In contrast, the combination of C8 increased the levels of PGC1α, DRP1, and MFN2. βHBA increased the levels of PGC1α and MFN2 but relatively decreased DRP1. To examine mitochondrial maturity, we examined the protein levels of leucine zipper and EF-hand containing transmembrane protein 1 (LETM1), a mitochondrial inner membrane marker, and translocase of outer mitochondrial membrane 20 (TOM20), an outer membrane marker. HMGB1 decreased LETM1 and increased TOM20, and the LETM1/TOM20 ratio decreased to 40%. In contrast, the combined use of C8 or βHBA increased LETM1 levels above normal and partially restored TOM20 levels. As a result, the LETM1/TOM20 ratio recovered to 83% of normal in C8 and 68% in βHBA. The excess inner membrane suggests cristae formation. Such changes in LETM1 and TOM20 were examined with three inflammatory cytokines (Figure 5B). IL6, TNFα, and HMGB1 all decreased LETM1, increased TOM20, and decreased the LETM1/TOM20 ratio. Among them, HMGB1 showed the strongest change. When mitochondrial morphology was examined, the mitochondrial long/short diameter ratio was decreased to 30% by HMGB1 (Figure 5C). In contrast, the concomitant use of C8 normalized the mitochondrial long/short-diameter ratio. However, we observed no significant recovery for βHBA. Thus, C8 exhibited a protective effect against HMGB1-induced mitochondrial damage, whereas the effect of βHBA was only partial compared to C8.

## 3. Discussion

In the present study, C8 showed the strongest mitochondrial protective effect among the three MCFAs and reduced cancer-induced myocardial damage. Additionally, among the three inflammatory cytokines, HMGB1 caused the most significant myocardial damage, accompanied by mitochondrial inhibition.

Our data showed that C8 had a stronger myocardial protective effect than C10 and C12 by improving mitochondrial dysfunction. We previously investigated the effect of C12 on cancer-induced myocardial damage. However, C12 alone did not sufficiently improve myocardial damage, and an improvement was observed when combined with glucose loading [11].

A study comparing C8, C10, and C12 reported that C12 alone reduced the risk of colorectal cancer [27]. Patients with breast cancer show decreased blood levels of C8 and C12 and increased levels of C10 [28]. While C8, C10, and C12 all reduce ROS levels during H_2_O_2_ treatment, nuclear factor erythroid 2-related factor 2 (Nrf2) activation was the strongest C12 [29]. The metabolism of MCFAs is enhanced by C8 and C10 during ketone body formation, whereas C12 promotes triacylglycerol formation [30]. C8 is rapidly and quantitatively altered to acetyl-CoA by β-oxidation and converted into ketone bodies, intermediates of TCA cycle, and glucogenic amino acids but is not readily assimilated into triglycerides [31].

The C8 diet induces AMP-activated protein kinase (AMPK) activation, resulting in a significant increase in PGC1a gene expression and protein levels [30]. AMPK is necessary for normal mitochondrial function in the heart and maintains the MCFA oxidation capacity [32]. These findings suggest that C8 is more effective than C10 or C12 in providing mitochondrial energy substrates through rapid metabolism and promoting mitochondrial biogenesis.

MCFAs are the main components of a ketogenic diet because they efficiently generate ketone bodies [26]. In the present study, we observed that C8 also increased plasma ketone body concentrations. Ketone bodies are used as metabolic substrates not only in the brain but also in other organs, such as the skeletal muscle and heart [33]. βHBA, the main component of ketone bodies, promotes the maintenance of myocardial function in acute myocardial infarction by promoting autophagy and preserving mitochondrial volume, function, and membrane potential [34,35]. βHBA also increases the nuclear levels of forkhead box protein (FOX)O1, FOXO3a, and PGC1α through the activation of sirtuin 2 (SIRT2) and AMPK activation to stimulate autophagy, mitophagy, and mitochondrial biogenesis [36]. However, in our study, βHBA was less effective than C8 in improving HMGB1-induced mitochondrial and myocardial tube damage. Ketone bodies are beneficial for mitochondrial repair in aging hearts; however, in heart failure, MFN2 and DRP1 expression is reduced by nearly 50%, impairing the fusion–fission process; thus, ketone bodies reduce mitophagy [37]. MCFAs have a stronger effect on improving cellular energy metabolism than ketone bodies through mitochondrial biogenesis [38]. These findings suggest that ketone bodies partially contribute to the myocardial-protective effects of C8. Therefore, future studies should examine the effects of MCFAs on miRNAs.

Our results revealed that, among IL6, TNFα, and HMGB1, HMGB1 exerted stronger mitochondrial impairment effects, which led to myocardial damage. In particular, HMGB1 reduced the mitochondrial inner membrane/outer membrane ratio, suggesting shortening of the mitochondrial cristae. Electron microscopy is required for morphological confirmation in future studies. LETM1 is a mitochondrial inner membrane marker required for maintaining mitochondrial morphology and cristae structure; thus, it is functionally important in regulating mitochondrial homeostasis [39]. Since the electron transport system is present in the cristae, the cristae represent the functional structure of mitochondria; however, these dynamic compartments change due to stress [40]. Although the mechanism underlying the effect of HMGB1 on LETM1 is unclear, LETM1 is affected by epigenetic signals [41], thus warranting further investigation in future studies.

Mitochondrial impairment promotes inflammatory cytokine expression and secretion [42,43]. In contrast, inflammatory cytokines damage mitochondria and increase ROS generation [44]. Inflammatory cytokines decrease mitochondrial respiration and PGC1α expression and increase p38 mitogen-activated protein kinase phosphorylation [45,46]. In the present study, HMGB1 suppressed autophagy and mitochondrial biogenesis and increased mitochondrial ROS levels in myocardial tubes.

In contrast, C8 ameliorated HMGB1-induced mitochondrial and myocardial tube damage. C8 suppresses inflammatory cytokine production and downregulates TLR4, myeloid differentiation primary response gene 88 (MyD88), nuclear factor-κB (NFκB), TNFα, and inhibitor of nuclear factor kappa-B kinase subunit α/β (IKBKB) mRNA and protein expression [47]. TLR4 is a receptor for HMGB1, and NFκB is a common intracellular signal for TLR4, which is a receptor for HMGB1, and RAGE [47]. Thus, C8 may improve myocardial damage by suppressing HMGB1 signaling [47].

In the present study, all three MCFAs suppressed peritoneally disseminated cancer in a mouse model of cachexia. We also previously reported the antitumor effects of C12 [11,48,49]. C12 promotes oxidative phosphorylation (OXPHOS) as a substrate for mitochondrial energy metabolism. Cancer cells show mitochondrial dysfunction and reduced quality control; therefore, OXPHOS promotion enhances ROS production and induces cell death [48,49,50]. In addition, C12 suppresses cell proliferation and invasion via the GPR120 G protein-coupled fatty acid receptor [51]. MCFAs reportedly inhibit breast, colon, and skin cancers [28,52,53]. Because of these antitumor effects, MCFAs may be useful for treating cancer-induced myocardial damage.

MCFAs may be effective against neurodegeneration in Alzheimer’s disease and cancer growth and metastasis, as well as the prevention of insulin resistance in type 2 diabetes [38]. Our research has shown that, among MCFAs, C8 has excellent mitochondrial biogenesis and maturation-promoting abilities and may be useful not only for the cancer-related myocardial disorders examined in the present study but also for myocardial disorders, such as ischemic heart diseases and chronic heart failure, as well as chronic kidney and neurodegenerative diseases induced by mitochondrial disorders.

## 4. Materials and Methods

### 4.1. Cell Culture

The CT26 mouse colon cancer cell line was gifted by Professor I. J. Fidler (MD Anderson Cancer Center, Houston, TX, USA). These cells were cultured in Dulbecco’s Modified Eagle Medium (DMEM; Wako Pure Chemical Industries, Ltd., Osaka, Japan) supplemented with 10% fetal bovine serum (Sigma-Aldrich Chemical Co., St. Louis, MO, USA). Embryonic rat heart-derived H9c2 cardiomyoblasts were purchased from the American Type Culture Collection (Manassas, VA, USA) and cultured in DMEM with 10% fetal bovine serum (Sigma-Aldrich).

### 4.2. Reagents

C8 (FUJIFILM Wako Pure Chemical Co., Osaka, Japan), C10 (FUJIFILM Wako Pure Chemical Co., Osaka, Japan), C12 (Tokyo Chemical Industry Co., Ltd., Tokyo, Japan), mouse IL6 (R&D Systems, Inc., Minneapolis, MN, USA), mouse TNFα (R&D Systems, Inc., Minneapolis, MN, USA), HMGB1 (BioLegend Inc., San Diego, CA, USA), and βHBA (Thermo Fisher Scientific Inc., Waltham, MA, USA) were purchased.

### 4.3. Cell Treatment

For cardiomyotube differentiation, H9c2 cardiomyoblasts were treated with differentiation medium (1% FBS, retinoic acid 1 μM in DMEM) for 48 h. After that, cardiomyotubes were treated with MCFAs or cytokines for 48 h. Treatment concentrations are 0.25 mM for C8, 2 mM for βHBA, 10 pg/mL for IL6, 100 pg/mL for TNF-α, and 100 ng/mL for HMGB1.

### 4.4. Animals

Five-week-old male BALB/c mice were purchased from SLC (Shizuoka, Japan) and maintained in a pathogen-free animal facility under a 12 h/12 h light/dark cycle in a temperature-controlled (22 °C) and humidity-controlled environment, in accordance with the institutional guidelines approved by the Committee for Animal Experimentation of Nara Medical University, Kashihara, Japan, following the current regulations and standards of the Japanese Ministry of Health, Labor, and Welfare (approval nos. 12924 and 13106, 2 August 2021). The animals were acclimated to their housing for seven days before the start of the experiment. The mice were fed a CE-2 diet containing 5% crude fat mainly derived from soybean oil (CLEA Japan, Inc., Tokyo, Japan). MCFA diets were prepared by mixing the control diet (CE-2) with C8, C10, or C12 MCFA at 2% (*w*/*w*). Diets were administered ad libitum, and intake was measured over time. Nutritional information of each diet is indicated in Table 1. Calorie intake (Figure 1B left) was determined by adding up the calories in each diet (Table 1) to the food intake (Figure 1B right) and dividing by the number of mice and the number of days administered.

To measure tumor weight, the mice were euthanized via aortic blood removal under sevoflurane anesthesia (Maruishi Pharmaceutical Co., Ltd., Osaka, Japan). The peritoneal tumors were then dissected from the intestine, mesenterium, diaphragm, and abdominal wall, and non-tumoral tissues were grossly removed.

**Figure 6:**
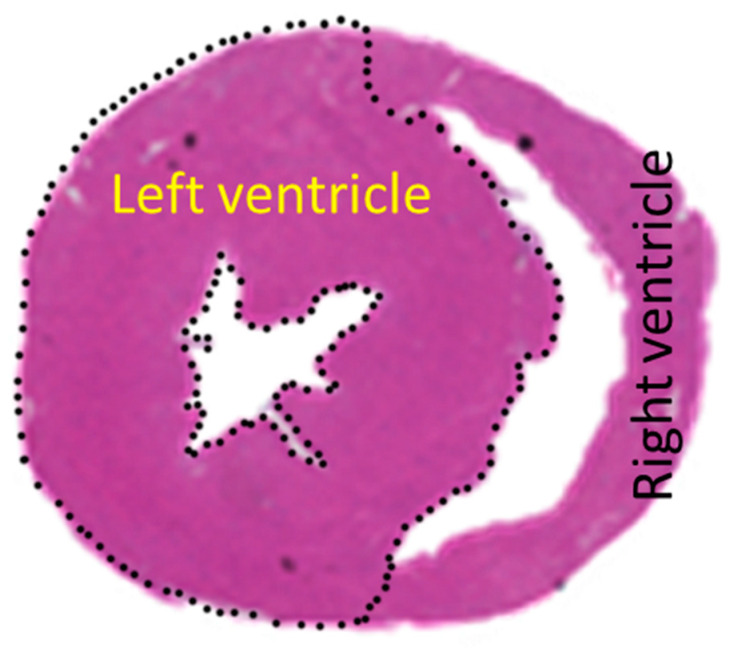
Left ventricular region determined. The left ventricle was dotted and distinguished from the right ventricle. The area was then calculated by dividing it into the HE-stained ventricular wall and the blank ventricular cavity.

After euthanasia, the heart was excised, weighed, and then divided into two parts at 2/5 from the apex of the heart. The upper part was used for histological analysis, while the lower part was used to analyze protein expression. The left ventricle was defined as that plane, excluding the right ventricular portion (Figure 6), and the image was imported into a computer, and the area was calculated using NIH ImageJ software (version 1.52, Bethesda, MD, USA). The border between the right and left ventricles in the ventricular septum was determined via a microscopic examination of the myocardial fiber runs.

To prepare the skeletal muscles, the QCM was cut at the muscle end on the upper edge of the patella, peeled from the femur, and separated at the muscle origin on the frontal surface of the anterior lower iliac spine. The excised QCM was weighed immediately, avoiding drying, and then stored at −80 °C.

### 4.5. Histological Analysis

The myocardial tissues were fixed in 4% paraformaldehyde, dehydrated, and embedded in paraffin. After slicing the block to 3 μm, hematoxylin and eosin staining was performed to observe the morphology. In the cut-surface specimen, the left ventricular area was selected, as shown in Figure 6. The image was scanned into a computer, and areas were calculated using NIH ImageJ software (version 1.52, Bethesda, MD, USA).

### 4.6. Protein Extraction

The lower part of the excised heart was stored at −80 °C, and the tissue was pulverized while still frozen to remove tendons and fascia. The muscle tissue was then washed with cold phosphate-buffered saline and pelleted using a sonicator (QSONICA, WakenBtech Co., Ltd., Kyoto, Japan). Whole-cell lysates were prepared as previously described, using radioimmunoprecipitation assay (RIPA) buffer with 0.1% sodium dodecyl sulfate (SDS) (Thermo Fisher Scientific, Tokyo, Japan). Protein assays were performed using a Protein Assay Rapid Kit (Wako Pure Chemical Corporation, Osaka, Japan). Extracted SDS-solubilized proteins were used for ELISA and Western blotting.

### 4.7. Enzyme-Linked Immunosorbent Assay (ELISA) and Colorimetric Assay

ELISA kits were used to measure the concentrations of MYL1, HMGB1, mouse TNFα, mouse IL6, 4HNE, and βHBA (Table 2). The assays were performed using whole-cell lysates, according to the manufacturer’s instructions.

### 4.8. Western Blotting

Lysates (20 μg) were subjected to immunoblot analysis using sodium dodecyl sulfate–polyacrylamide gel electrophoresis (SDS-PAGE; 12.5%), followed by electrotransfer onto nitrocellulose filters. The filters were incubated with primary antibodies (Table 2), followed by peroxidase-conjugated IgG antibodies (Medical and Biological Laboratories). Anti-β-actin antibody was used to assess the levels of protein loaded per lane (Santa Cruz Biotechnologies, Santa Cruz, CA, USA). The immune complex was visualized using an Enhanced Chemiluminescence Western-blot detection system (Amersham, Aylesbury, UK).

### 4.9. Reverse-Transcription Polymerase Chain Reaction (RT-PCR)

Total RNA (1 μg) was used to synthesize complementary DNA (cDNA) using the ReverTra Ace quantitative PCR (qPCR) RT kit (Toyobo, Osaka, Japan). PCR was performed according to the manufacturer’s instructions. The PCR products were electrophoresed on 2% agarose gels and visualized using ethidium bromide. The primer sets are listed in Table 2. The primers were synthesized by Sigma-Aldrich (Ishikari, Japan).

### 4.10. Mitochondrial Imaging

Mitochondrial function was examined using fluorescent probes (Table 2). The cells were incubated with the probes for 30 min at 37 °C and then imaged using a BZ-X710 all-in-one fluorescence microscope (KEYENCE, Osaka, Japan). We used MitoROS for the detection of mitochondrial superoxide (10 μM), Dihydrorhodamine 123 (DHR) for mitochondrial hydrogen peroxide (10 μM), MitoBright LT Green for mitochondrial volume (100 μM), tetramethylrhodamine, ethyl ester (TMRE) for MMP (200 nM), CellMeter for hydroxyl radical (•OH) (10 μM), and Lipefluo for lipid peroxide (10 μM). The assays were performed according to the manufacturer’s instructions [54,55,56,57]. Mitochondrial morphology was examined using MitoBright LT Green staining and observed on the BZ-X710 microscope with a 100× objective lens.

### 4.11. Statistical Analysis

Statistical significance and statistical differences were calculated using analysis of variance with Bonferroni correction in InStat software (version 3.0; GraphPad Software, Inc., La Jolla, CA, USA). Data are expressed as the mean ± standard deviation of three independent experiments. Statistical significance was set at *p* < 0.05 (two-sided).

## Figures and Tables

**Table 1:** Nutritional information.

	Diet			
	Control	C8	C10	C12
MCFA (%)	0	2	2	2
Moisture (%)	7.95	7.791	7.791	7.791
Crude protein (%)	25.06	24.5588	24.5588	24.5588
Crude fat (%)	4.78	4.6844	4.6844	4.6844
Crude fiber (%)	4.91	4.8118	4.8118	4.8118
Crude ash (%)	6.88	6.7424	6.7424	6.7424
Nitrogen-free extract (%)	50.42	49.4116	49.4116	49.4116
Energy (Kcal)	345	356.1	356.1	356.1

**Table 2:** Primer sets, antibodies, enzyme-linked immunosorbent assay (ELISA) kits, and detection kits.

**Primer Set**			
**Gene Symbol**	**GenBank ID**	**Forward (5** **′–3** **′)**	**Reverse (5** **′–3** **′)**
Myogenin		CTTCTCCCTCAGTGTGGCTG	ACCAGGAGCCCCACTTCTAT
Myomesin		ACTGCTCACCGGTGGTTAAG	GTGTTCCGGGTTTTGTCAGC
TroponinT		GAGCCTCGATCAGAGTCTGC	TGGAGGAGGAGGATGGTGAG
β-actin		CGGAACCGCTCATTGCCGAT	ACCGAGCGTGGCTACAGCTT
**Antibody**			
**Target**	**No**	**Company**	
PGC1α	#2178S	Cell Signaling Technology, Danvers, MA, USA	
LETM1	16024-1-AP	Proteintech, Tokyo, Japan	
TOM20	11802-1-AP	Proteintech, Tokyo, Japan	
PINK1	23274-1-AP	Proteintech, Tokyo, Japan	
Parkin	#4211	Cell Signaling Technology, Danvers, MA, USA	
LC3	PM036	Medical & Biological Laboratories, Tokyo, Japan	
MYH6	22281-1-AP	Proteintech, Tokyo, Japan	
MYH7	22280-1-AP	Proteintech, Tokyo, Japan	
Beclin1	11306-1-AP	Proteintech, Tokyo, Japan	
β-actin	sc-47778	Santa Cruz Biotechnologies, Santa Cruz, CA, USA	
**ELISA**			
**Protein**	**Cat No**	**Company**	
TNF-α	MTA00B	R&D Systems, Inc., Minneapolis, MN, USA	
HMGB1	326078738	Shino-Test Co., Kanagawa, Japan	
4HNE	CSB-E13412m	Cusabio Biotech Co., Houston, TX, USA	
MYL1	RK07660	Abclonal, Woburn, MA, USA	
Detection kit			
**Target**	**Product and Cat No**	**Company**	
TMRE	TMRE, 9103	ImmunoChemistry Technologies, LLC., Minneapolis, MN, USA	
Mitochondria	MitoBright LT Green. 346-92061	Dojindo Laboratories, Kumamoto, Japan	
Mitochondrial superoxide	MitoROS, 16052	AAT Bioquest, Inc., Pleasanton, CA, USA	
Mitochondrial hydrogen peroxide	DHR123, 85100	Cayman Chemical, Ann Arbor, MI, USA	
Hydroxyl radical	CellMeter, 16055	AAT Bioquest, Inc., Pleasanton, CA, USA	
Lipid peroxide	Lipefluo, 1448846-35-2	Dojindo Laboratories, Kumamoto, Japan	
β-Hydroxybutyrate	700740	R&D Systems, Incorporated, Minneapolis, MN, USA	

## Data Availability

Data is contained within the article.

## Abbreviations

MCFA, medium-chain fatty acid; C8, caprylic acid; C10, capric acid; C12, lauric acid; HMGB1, high mobility group box-1; CTRCD, cancer therapeutic-related cardiac dysfunction; IL, interleukin; TNF, tumor necrosis factor; RAGE, advanced glycation end products; TLR, toll-like receptor; QCM, quadriceps femoris muscle; SDS-MYL1, sodium dodecyl sulfate-soluble myosin light chain-1; 4-HNE, 4-hydroxynonenal; βHBA,β-hydroxybutyric acid; ROS, reactive oxygen species; MMP, mitochondrial membrane potential; MYH, myosin heavy chain; LC3, autophagy-related proteins light chain 3; PINK1, PTEN-induced kinase 1; DRP1, dynamin related protein-1; MFN2, mitofusin-2; PGC1α, peroxisome proliferator-activated receptors gamma coactivator-1 alpha; LETM1, leucine zipper and EF-hand containing transmembrane protein 1; Nrf2, nuclear factor erythroid 2-related factor 2; AMPK, AMP-activated protein kinase; FOX, forkhead box protein; SIRT, sirtuine; MyD88, myeloid differentiation primary response gene 88; IKBKB, inhibitor of nuclear factor kappa-B kinase subunit α/β; NFκB, nuclear factor-κB; OXPHOS, oxidative phosphorylation.
